# An interpretable machine learning model for predicting the risk of in-hospital death among ICU patients with traumatic brain injury

**DOI:** 10.3389/fneur.2026.1987337

**Published:** 2026-10-29

**Authors:** Yunfan Ji, Wei Hu, Xia Li

**Affiliations:** 1 Department of Nursing, First Affiliated Hospital of Jinzhou Medical University, Jinzhou, China; 2 Department of Nursing, People's Hospital of Shangrao, Shangrao, Jiangxi, China; 3 First Affiliated Hospital of Jinzhou Medical University, Jinzhou, Liaoning, China

**Keywords:** in-hospital mortality, machine learning, random forest, risk prediction model, SHAP, traumatic brain injury

## Abstract

**Background:**

Traumatic brain injury (TBI) is one of the leading causes of death and disability; early and accurate prediction of a patient’s risk of in-hospital mortality is crucial for optimizing treatment and improving outcomes.

**Objective:**

The aim of this study is to develop and validate a machine-learning-based model for predicting the risk of in-hospital mortality among ICU patients with traumatic brain injury, and to use interpretable methods to identify key predictive factors and assess their clinical utility.

**Methods:**

Clinical data were retrospectively collected from patients with TBI admitted to the intensive care units (ICUs) of two Grade A, Level 3 hospitals between January 2022 and December 2025; After excluding cases of death due to causes other than TBI and those who were already brain-dead on admission, a total of 874 patients were ultimately included in the study. This was a 24-h milestone prediction study; the predictive model established is applicable only to patients with traumatic brain injury who remained alive 24 h after admission to the ICU. The primary outcome was in-hospital death due to traumatic brain injury; excluding deaths from causes other than traumatic brain injury and cases where brain death had already been confirmed on admission, a total of 381 cases experienced this outcome, representing an incidence rate of 43.59 per cent. Prior to analysis, the dataset was randomly divided into a training group (*n* = 613) and a testing group (*n* = 261). Lambda 1-se standard LASSO regression was applied solely to the training set to identify predictive features, resulting in the selection of 17 candidate variables. Seven machine learning algorithms were developed: logistic regression, random forest, XGBoost, artificial neural networks, decision trees, support vector machines and naive Bayes. Model performance was evaluated using the area under the receiver operating characteristic curve (AUC), recall, accuracy, F1 score, precision and negative predictive value, with 95 per cent confidence intervals calculated for all metrics; interpretability analysis and model pruning were performed using the SHAP method.

**Results:**

LASSO regression identified 17 relevant key features; the random forest model demonstrated good predictive performance, with a training set cross-validation AUC of 0.954. The simplified RF model constructed after feature dimensionality reduction included 11 key predictive variables, with AUC on the test set was 0.924 (95% CI: 0.848–0.960).

**Conclusion:**

The random forest-based predictive model demonstrated high predictive performance; the simplified model strikes a balance between performance and clinical interpretability and can effectively identify high-risk populations. Key features such as GCS and APACHE II can support early clinical risk stratification and personalized interventions, highlighting the potential of machine learning in the clinical management of TBI.

## Introduction

Traumatic brain injury (TBI) is a common and critical emergency in neurosurgery, characterized by sudden onset, severe condition, and high rates of disability and mortality. Globally, approximately 1.5 million people die from TBI each year, and millions more require emergency treatment (1, 2); The in-hospital mortality rate for patients with moderate-to-severe TBI can reach 20 to 40%, placing a heavy medical and financial burden on society and families. TBI refers to brain tissue damage caused by acute trauma (3) and has become a major medical issue affecting both high-income (4, 5) and low- and middle-income countries (6) worldwide, significantly impacting patients’ survival outcomes, functional prognosis, and quality of life (7–9). The clinical course of patients with TBI is influenced by multiple factors, including age, severity of injury, complications, and therapeutic interventions; consequently, there is significant individual variation in the risk of in-hospital mortality. Early and accurate identification of patients at high risk of death, followed by targeted interventions, is key to reducing mortality and improving clinical outcomes. Similarly, the timely identification of TBI patients at high risk of death and severe adverse outcomes is crucial for optimizing the allocation of trauma care resources and improving patient outcomes (10). Prognostic modeling can predict various outcomes of TBI based on patients’ clinical data, providing important support and objective evidence for clinical decision-making (11).

Currently, most clinical tools used to assess the prognosis of TBI are single-scale systems. The Glasgow Coma Scale (GCS) is a classic tool for evaluating the degree of impaired consciousness in patients with brain injury (12), but it focuses solely on neurological function and lacks a comprehensive assessment of overall physiological status; Although the Acute Physiology and Chronic Health Evaluation II (APACHE II) (13) score can reflect a patient’s overall organ function and disease severity, it lacks specificity for assessing the prognosis of brain injury. In terms of assessing organ dysfunction, the Sequential Organ Failure Assessment (SOFA) score (14) effectively reflects the severity of multiple organ failure by evaluating six major systems—respiratory, coagulation, hepatic, circulatory, neurological, and renal—and is closely associated with mortality in critically ill patients. However, the progression of TBI is often accompanied by complex systemic pathophysiological changes. Changes in physiological parameters such as systolic blood pressure (SBP), diastolic blood pressure (DBP), heart rate (HR), and lactate (Lac) that occur in the early stages of injury can provide important insights for prognostic assessment (15–17). A single scoring system or isolated physiological indicators often fail to fully capture the complexity of traumatic brain injury; their predictive power is limited, making it difficult to meet the need for precise clinical prognosis assessment. In an intensive care setting, the early and accurate identification of high-risk patients and the implementation of interventions are key to improving patient outcomes. Early identification of patients at high risk of death is crucial for the rational allocation of medical resources and the timely implementation of active interventions such as intracranial pressure monitoring and craniectomy, thereby improving patient outcomes (18).

Machine learning (ML) has become an important tool for clinical predictive modeling and offers significant advantages in uncovering complex nonlinear relationships within medical data (19–21). With the continuous advancement of healthcare informatization, electronic health records have accumulated vast amounts of multidimensional clinical data, providing a data foundation for building precise disease risk prediction models. Machine learning technologies can effectively uncover latent correlations within high-dimensional data and show great promise for application in various clinical fields, including intensive care. Numerous studies have demonstrated that machine learning performs exceptionally well in predicting outcomes for various neurosurgical conditions, including TBI, epilepsy, and brain tumors (22–24). To overcome the limitations of traditional statistical models, previous studies have applied machine learning algorithms to predict the prognosis of patients with traumatic brain injury (25, 26). Among adults with TBI, these models also demonstrate superior predictive performance compared to various traditional predictive models (27, 28); even among pediatric TBI patients, machine learning models outperform traditional statistical methods and CT classification systems in prognostic assessment (29).

Although traditional machine learning methods have demonstrated strong performance in risk prediction, their “black-box” nature makes it difficult to explain the mechanisms underlying key risk factors and the prediction process. This not only undermines clinical confidence in model decisions but also, to some extent, limits their acceptance and widespread adoption in clinical practice (30–32). However, these limitations can be addressed using SHapley Additive Explanations (SHAP), a system that illustrates the extent to which each feature contributes to a single prediction, thereby making the model more transparent (33). By quantifying the contribution of each feature to the model’s predictive outcomes using the SHAP algorithm, this approach reveals the underlying mechanisms linking features to outcomes, effectively addressing the “black box” problem and laying the groundwork for the clinical translation of machine learning models.

Although significant progress has been made in the application of ML to the prediction of TBI, several key gaps remain. First, most existing studies focus on advanced imaging modalities or molecular biomarkers, with limited research combining these approaches with scoring systems. Second, many studies have failed to adequately address the risk of data leakage resulting from improper splitting of training and test sets, which may lead to overly optimistic performance estimates. Third, model evaluation is often limited to AUC, neglecting a comprehensive assessment of sensitivity, specificity, and clinical utility metrics. Fourth, although SHAP analysis has the potential to enhance clinical interpretability, its systematic integration with feature-selection machine learning models has not yet been fully explored. This study integrates multidimensional clinical data from TBI patients to construct seven machine learning models for predicting in-hospital mortality. Using a rigorous data splitting strategy to prevent information leakage, we conducted a comprehensive performance evaluation to identify the optimal model and applied the SHAP method to interpret the model. Simultaneously developing simplified, interpretable predictive models. This study aims to identify, at an early stage, high-risk groups for in-hospital mortality among patients with traumatic brain injury (TBI) in clinical ICUs, the identification of key factors influencing prognosis, and the formulation of individualized intervention strategies. The development of such models fills a gap in existing research and provides a valuable support tool for emergency medical decision-making (34).

## Materials and methods

### Study population

We retrospectively collected clinical data on patients with TBI admitted to the intensive care units (ICUs) of two Grade A Level 3 hospitals between January 2022 and December 2025. In this study, the sample size was determined by comprehensively considering model complexity and the volume of clinically available data; the sample size design was based on the classic Events Per Variable (EPV) criterion established by Peduzzi et al. (35). Revised guidelines for the event risk ratio recommend that the EPV for nonlinear models should reach 15 (36). The training set for this study comprised a total of 267 outcome events (in-hospital death). Initially, 55 candidate clinical variables were included in the modeling process, at which point the EPV was approximately 4.9; this stage was used solely for variable selection via Least Absolute Shrinkage and Selection Operator (LASSO) regression and did not involve the construction of the final model; After preliminary screening using LASSO regularization, 17 variables were retained for the random forest modeling process, with the EPV rising to 15.7; following SHAP feature importance ranking and stepwise pruning optimization, 11 variables were ultimately included in the optimal model, with an EPV of 24.3. To mitigate the potential risk of overfitting caused by a low EPV during the initial variable selection phase, this study employs rigorous regularization techniques and conducts model optimization using cross-validation. This study collected clinical data from a total of 1,162 patients with TBI; after screening based on inclusion and exclusion criteria, 874 patients were ultimately included in the study. Inclusion Criteria: (1) Meets the diagnostic criteria for TBI (confirmed by CT or MRI, such as cerebral contusion, intracranial hemorrhage, skull fracture, and traumatic subarachnoid hemorrhage); (2) Age ≥18 years; (3) Glasgow Coma Scale (GCS) score ≤14; (4) Complete clinical data, including baseline information, laboratory tests, imaging studies, treatment interventions, and follow-up information required for this study; (5) Received standard treatment after admission. Exclusion criteria: (1) Concurrent severe underlying diseases of vital organs such as the heart, liver, kidneys, or lungs, or malignant tumors; (2) Concurrent irreparable severe trauma to other body parts; (3) Brain death or irreversible failure of vital signs at the time of admission; (4) Missing clinical data; (5) Death during hospitalization due to causes unrelated to TBI. This study followed a 24-h landmark prediction design. Patients who died within 24 h after ICU admission were excluded from model development and validation, as complete 24-h predictor variables could not be obtained for these patients. Accordingly, the prediction model presented in this study applies exclusively to patients who are alive at 24 h after ICU admission.

### Data collection and variable definition

Clinical data were collected within 24 h of hospital admission via the electronic medical record system. A total of 55 potential predictor variables were included, covering five dimensions: demographic characteristics: age, sex, medical history, and medication history, including stroke, hypertension, diabetes, atrial fibrillation, etc.; Clinical characteristics: A series of clinical parameters, including the GCS score, APACHE II score, SOFA score, FOUR score, Pupil Size and Reflexes (with normal pupil size and the presence of a light reflex serving as the reference group), blood pressure, pulse, heart rate, respiration, and body temperature; Laboratory parameters: white blood cell count, platelet count, hemoglobin, prothrombin time, activated partial thromboplastin time, blood glucose, serum sodium, serum potassium, lactate, etc.; Therapeutic interventions: mode of respiratory support, use of vasoactive medications; Imaging characteristics: type of imaging study, hemorrhage volume, infarct extent, degree of midline shift, brain herniation, and ventricular hemorrhage; Prognosis indicators: in-hospital mortality, defined as death occurring during hospitalization due to traumatic brain injury and its complications.

All predictive factors were restricted to baseline data collected within 24 h of admission to the ICU: ① Neurological function scores (GCS, FOUR) were assessed for the first time within 24 h of admission; for sedated patients, these were determined by combining records of consciousness at the time of injury, pre-hospital assessment and pain responses upon admission, with priority given to the score from the patient’s last conscious state prior to medication administration to minimize the impact of sedation. ② For organ function scoring, APACHE II uses the worst physiological parameters recorded within 24 h of admission to the ICU, whilst SOFA assessed the worst organ function levels within 24 h of ICU admission; both calculations were completed at the baseline time point. ③ Laboratory parameters are based on the results of the first peripheral venous blood test performed within 24 h of admission. ④ Treatment-related variables, such as respiratory support and vasoactive drugs, are defined as whether the relevant treatment had been initiated within 24 h of admission; these serve as surrogate indicators of baseline disease severity and do not include treatment information obtained after the baseline period.

### Data preprocessing

The collected raw data underwent standard preprocessing to improve model stability and predictive performance: ① Handling of missing values: This study employed a rigorous data collection and quality control protocol, excluding all cases with missing values for core clinical variables such as age, GCS score, pupillary light reflex, and APACHE II score, to ensure that the final dataset included in the analysis contained no missing values. ② Outlier handling: The interquartile range (IQR) method was used to screen for extreme outliers in continuous variables. Specifically, values falling outside the range of Q1–1.5 × IQR or Q3 + 1.5 × IQR were considered potential extreme outliers. After individual verification, only those values confirmed to be data entry errors or outside the clinically plausible range were excluded. It should be emphasized that this method was used in this study solely to identify and exclude extreme outliers, rather than to winsorize all continuous variables across their entire range. For markers such as lactate and procalcitonin, which exhibit significant individual variability among patients with severe TBI, high values within clinically reasonable ranges were retained to preserve predictive information regarding disease severity. ③ Data Split: Using stratified random sampling, the dataset is split into a training set and a test set in a 7:3 ratio. The training set is used for model development and parameter optimization. To prevent data leakage and ensure the integrity of model evaluation, the test set is strictly isolated throughout the feature selection process and is used solely for final model validation. Stratified sampling ensures that the proportion of in-hospital deaths in the training and testing sets matches that of the original cohort, thereby avoiding model bias caused by uneven distribution of outcomes. All data-dependent preprocessing steps, including outlier handling and standardization, were performed after the training/test split. The IQR thresholds and standardization parameters were estimated solely from the training set and applied unchanged to the test set.

### Feature screening

Feature selection was performed using only LASSO (Least Absolute Shrinkage and Selection Operator) regression, implemented via the glmnet package, on the training set. LASSO regularization uses 10-fold cross-validation to determine the optimal tuning parameter (λ) and applies the “lambda.1se” criterion, which selects a regularized model whose error falls within one standard deviation of the minimum cross-validation error, thereby promoting parsimony while maintaining predictive performance. Using in-hospital mortality as the dependent variable and the 55 preprocessed initial variables as independent variables, we identified 17 variables with non-zero coefficients and subsequently used these variables as input variables for subsequent machine learning models.

### Model development and parameter optimization

Using the features selected via LASSO regression as input and in-hospital mortality as the output, seven machine learning prediction models were developed on the training set: logistic regression (LR), support vector machines (SVM), decision trees (DT), artificial neural networks (ANN), naive Bayes (NB), XGBoost, and random forests (RF). All models were built using R software (version 4.4.3). To avoid overfitting and optimize predictive performance, hyperparameter tuning was performed on all models (see Supplementary materials); grid search combined with 10-fold cross-validation was used to systematically optimize the key hyperparameters of each model and identify the optimal combination of hyperparameters.

### Model performance evaluation

We conducted a comprehensive evaluation of the predictive performance of seven machine learning models on the test set, using the following four key evaluation metrics: ① Area under the receiver operating characteristic curve (AUC): Reflects the model’s discriminatory power; the closer the AUC is to 1, the better the model’s ability to distinguish between patients at high and low risk of death; ② Accuracy: Reflects the proportion of correctly predicted samples out of the total number of samples; ③ Recall: The proportion of in-hospital deaths correctly predicted by the model out of the total number of actual in-hospital deaths, also known as sensitivity; ④ F1-Score: The harmonic mean of precision and recall, which comprehensively reflects the model’s overall predictive performance. We also calculated three additional metrics—precision, negative predictive value (NPV), and specificity—to comprehensively evaluate the model’s classification performance: precision reflects the proportion of patients predicted by the model to die in-hospital who actually died; negative predictive value reflects the proportion of patients predicted by the model to survive who actually survived; and specificity reflects the model’s ability to correctly identify surviving patients. All binary classification metrics (recall, accuracy, precision, F1 score, and specificity) are calculated based on a uniform probability threshold of 0.5. This threshold was not optimized on the test set to avoid optimism bias and ensure comparability among models. At the same time, ROC curves were plotted for each model to visually compare their discriminatory capabilities; calibration curves were plotted for the final model to assess the consistency between the model’s predicted probabilities and the actual occurrence probabilities. The 95% confidence intervals for all metrics were calculated using the percentile method, derived through the bootstrap distribution method implemented in the pROC and caret packages, thereby providing a robust quantification of uncertainty. In a 10-fold cross-validation of the training set, after comprehensively considering the average AUC across cross-validation folds, calibration performance, and model interpretability, a random forest was ultimately selected as the final model for further optimization and feature selection. The test set was set aside separately and remained completely unused during the model selection process; it was used only for a one-time independent evaluation and descriptive comparison after the final model was determined, thereby ensuring the objectivity of the performance evaluation.

### Model interpretability analysis and simplified model construction

The model with the best performance was selected, and the SHAP method was used to conduct an interpretability analysis: (1) SHAP scores were calculated for each feature to quantify their contribution to the model’s predictive results; a SHAP summary plot was generated, and features were ranked by importance to identify the key features influencing in-hospital mortality among TBI patients; (2) a SHAP dependency plot was generated to analyze the nonlinear relationship between key features and the risk of in-hospital mortality. SHAP analysis is used solely to quantify the extent and direction of each feature’s contribution to the model’s predictions and to reflect the importance of the association between features and outcomes; it does not imply a causal relationship, nor can it be used to directly infer the efficacy of an intervention.

Based on the SHAP feature importance ranking results, this study employs a sequential backward pruning method to select the optimal feature subset: features are iteratively pruned in ascending order of importance, with the three least important features removed in each round. This step-size strategy enhances the efficiency of feature subset exploration, prevents minor performance fluctuations caused by the removal of individual features from interfering with the decision to stop, and balances pruning granularity with model performance stability. Each feature subset underwent 10-fold cross-validation on the training set; the out-of-fold prediction results were combined to construct ROC curves, and the DeLong test was employed to compare the paired ROC curves of adjacent feature subsets. Pruning employs a dual stopping criterion: if, compared with the previous feature subset, the relative decrease in cross-validation AUC exceeds 2 per cent, or if the DeLong test indicates a statistically significant difference (*p* < 0.05), the removed feature is deemed a key predictor and pruning is halted. This threshold is determined based on clinical evidence, balancing model simplicity with clinical value, resulting in only minimal loss of discriminatory performance whilst retaining core prognostic indicators. The entire feature selection and pruning process was conducted exclusively on the training set. The independent test set remained locked throughout and was not involved in any pruning decisions; it was used solely for a one-off independent evaluation of the final model, thereby ensuring unbiased results. The calibration curve was plotted using the rms package, and the Hosmer-Lemeshow test was performed to assess the model’s calibration; decision curve analysis was conducted using the rmda package to quantify the net benefit at different probability thresholds, thereby evaluating the model’s clinical utility. All modeling procedures adhere to the “Transparency Reporting of Individualized Prognosis or Diagnosis Multivariable Prediction Models” (TRIPOD) guidelines.

### Statistical analysis

Statistical analysis was performed using SPSS 27.0 and R software. Continuous variables are expressed as median (interquartile range) [M (IQR)], and intergroup comparisons were performed using the Mann–Whitney U test. Categorical variables are presented as frequencies (percentages) [n (%)], and comparisons between groups were performed using the chi-square test or Fisher’s exact test. *p* < 0.05 is considered statistically significant. Model performance was compared using the Delong test, with a significance level of *p* = 0.05 (see Figure 1).

**Figure 1:**
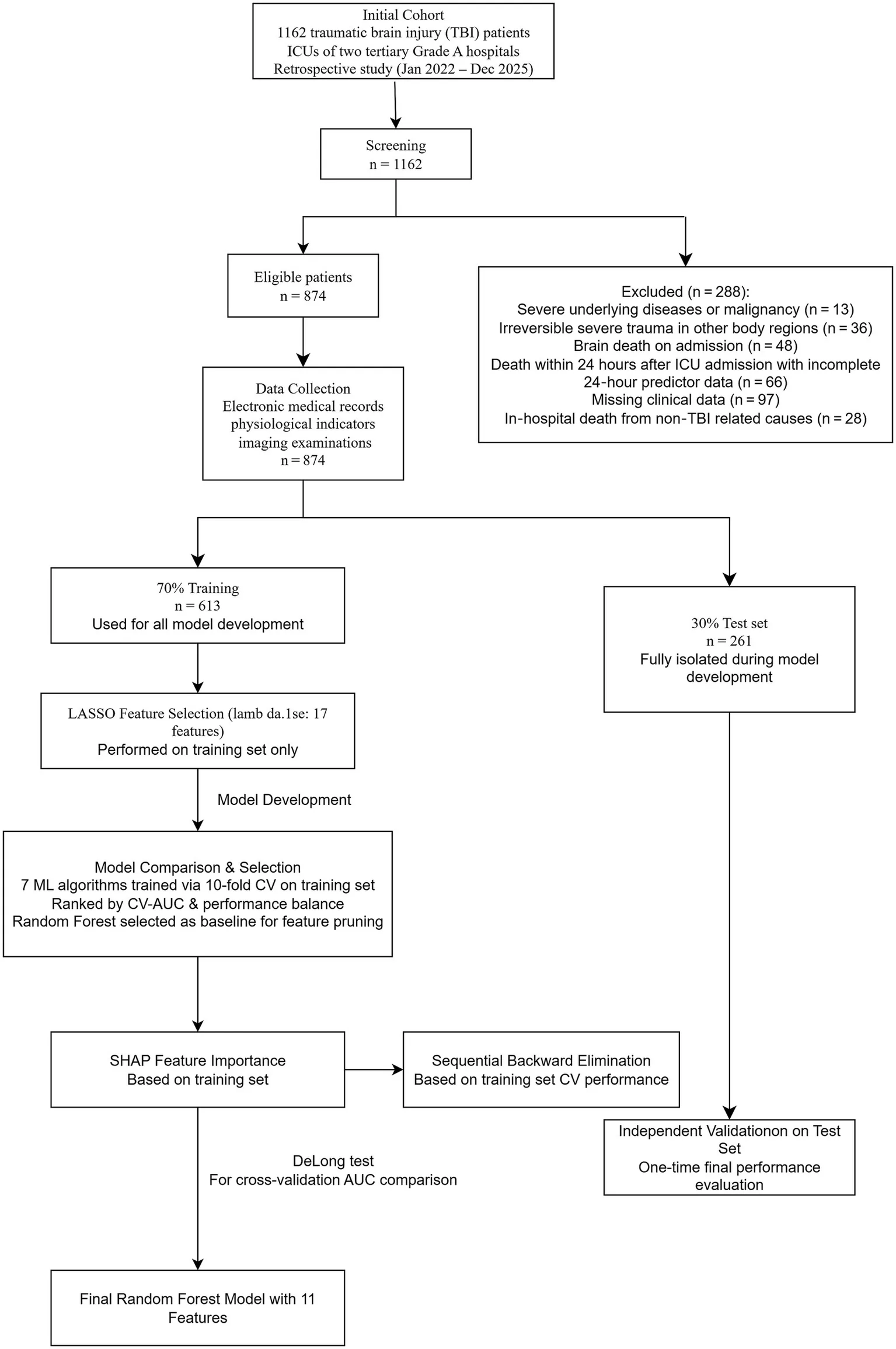
Study flowchart. This figure illustrates the patient selection, data collection, LASSO feature selection, model development and comparison, and SHAP-guided feature pruning processes.

## Results

### Demographic characteristics of the study population

This study collected clinical data from a total of 1,162 patients with traumatic brain injury. Following screening against inclusion and exclusion criteria, and based on a 24-h milestone prediction design, 874 patients who were still alive 24 h after admission to the ICU were ultimately included to form the study cohort. The cohort comprised 586 males (67.0%) and 288 females (33.0%); with a median age of 62 years; of these, 381 patients experienced the primary outcome (in-hospital death), resulting in an in-hospital mortality rate of 43.59%, whilst the remaining 493 patients survived their hospitalization. In this study, patients were categorized into a death group and a survival group based on their in-hospital survival status, and the clinical characteristics of the two groups were compared and analyzed. The results showed that, compared with the survival group, patients in the mortality group exhibited statistically significant differences (*p* < 0.001) in pupil size, light reflex, vasoactive agents, blood loss, midline shift, oxygen saturation, GCS score, FOUR score, APACHE II score, SOFA score, white blood cell count, prothrombin time, D-dimer, blood urea nitrogen, and lactate were all statistically significant (*p* < 0.001) (see Supplementary Table 1).

### Feature selection results

Feature selection was performed on the initial 55 variables using LASSO regression. Ultimately, 17 variables with non-zero coefficients were identified, namely: APACHE II score, GCS score, SOFA score, FOUR score, prothrombin time, lactate, procalcitonin, D-dimer, midline shift, pupil size, light reflex, diastolic blood pressure, albumin, oxygen saturation, blood loss, antihypertensive medication, and chloride levels (see Figure 2).

**Figure 2:**
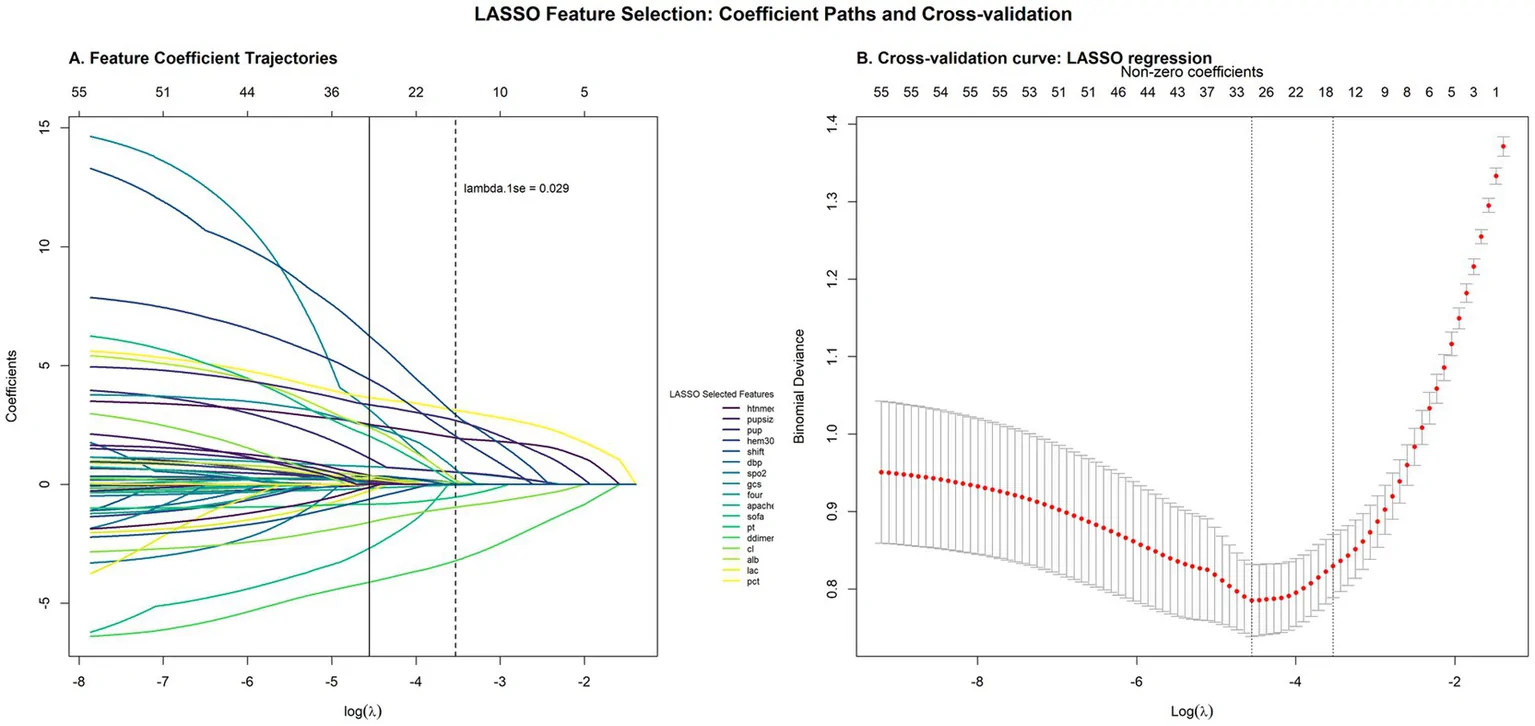
LASSO feature selection: coefficient paths and cross-validation. **(A)** Feature coefficient trajectories. Colored lines represent coefficient values of candidate features changing along log(*λ*). The vertical dashed line marks the optimal *λ* (lambda.1se = 0.029), and numbers on the top axis indicate the number of non-zero coefficients. **(B)** Cross-validation plot of binomial deviance against log(λ). The red dotted line shows binomial deviance, gray error bars represent standard error, and the vertical dashed line corresponds to the optimal *λ* for feature screening. The legend lists selected features and their color codes.

### Comparison of machine learning model performance

The algorithm selection in this study was based entirely on the results of 10-fold cross-validation on the training set; the test set was used solely for descriptive performance analysis after the final model was selected and was not involved in the model selection process at any stage to avoid data leakage.

Table 1 shows the predictive performance metrics of the seven machine learning models on the independent test set; the corresponding ROC curves are shown in Figure 3. The results show that all seven models demonstrated a certain ability to distinguish the risk of in-hospital mortality (all models had an AUC > 0.80). Among them, the Random Forest model had the highest AUC value of 0.923 (95% CI: 0.889–0.956), with a precision of 0.877, a recall of 0.842, and an F1 score of 0.857; followed by the XGBoost model, with an AUC of 0.917 (95% CI: 0.882–0.953); Logistic regression, as a traditional statistical model, had an AUC of 0.916 (95% CI: 0.882–0.950), outperforming support vector machines, artificial neural networks, Naive Bayes, and decision tree models; the decision tree model had the weakest discriminatory power, with an AUC of 0.835 and a recall of 0.763. Validation using the paired DeLong test on the test set’s predicted values showed that the differences in AUC between Random Forest and XGBoost (*p* = 0.898) as well as logistic regression (*p* = 0.639) were not statistically significant; the 95% confidence intervals for all three models overlapped extensively, indicating comparable overall discriminatory performance.

**Table 1:** Performance metrics (95% confidence interval) of machine learning models for predicting in-hospital mortality in patients with traumatic brain injury..

Model	AUC (95% CI)	Recall (95% CI)	Accuracy (95% CI)	F1 Score (95% CI)	Precision (95% CI)	NPV (95% CI)	Specificity (95% CI)
RF	0.923 (0.889–0.956)	0.842 (0.777–0.907)	0.877 (0.839–0.920)	0.857 (0.808–0.903)	0.873 (0.810–0.933)	0.881 (0.831–0.931)	0.905 (0.854–0.950)
XGBoost	0.917(0.882–0.953)	0.842(0.778–0.908)	0.870(0.828–0.908)	0.850(0.800–0.897)	0.857(0.793–0.918)	0.879(0.830–0.931)	0.891(0.838–0.938)
LR	0.916 (0.882–0.950)	0.895 (0.839–0.948)	0.805 (0.759–0.854)	0.800 (0.743–0.851)	0.723 (0.650–0.799)	0.900 (0.847–0.950)	0.735 (0.669–0.807)
SVM	0.905 (0.868–0.942)	0.886 (0.828–0.941)	0.854 (0.812–0.897)	0.842 (0.790–0.888)	0.802 (0.732–0.871)	0.904 (0.857–0.952)	0.830 (0.772–0.890)
ANN	0.855(0.809–0.900)	0.763(0.689–0.839)	0.785(0.739–0.835)	0.757(0.697–0.814)	0.750(0.675–0.829)	0.814(0.752–0.876)	0.803(0.739–0.865)
NB	0.854 (0.807–0.901)	0.728 (0.650–0.813)	0.789 (0.743–0.835)	0.751 (0.690–0.812)	0.776 (0.698–0.852)	0.799 (0.737–0.862)	0.837 (0.776–0.892)
DT	0.835 (0.780–0.890)	0.763 (0.682–0.841)	0.820 (0.774–0.862)	0.787 (0.729–0.844)	0.813 (0.740–0.887)	0.825 (0.765–0.880)	0.864 (0.805–0.919)

RF: Random Forest; LR: Logistic Regression; SVM: Support Vector Machine; ANN: Artificial Neural Network; NB: Naive Bayes; DT: Decision Tree.

**Figure 3:**
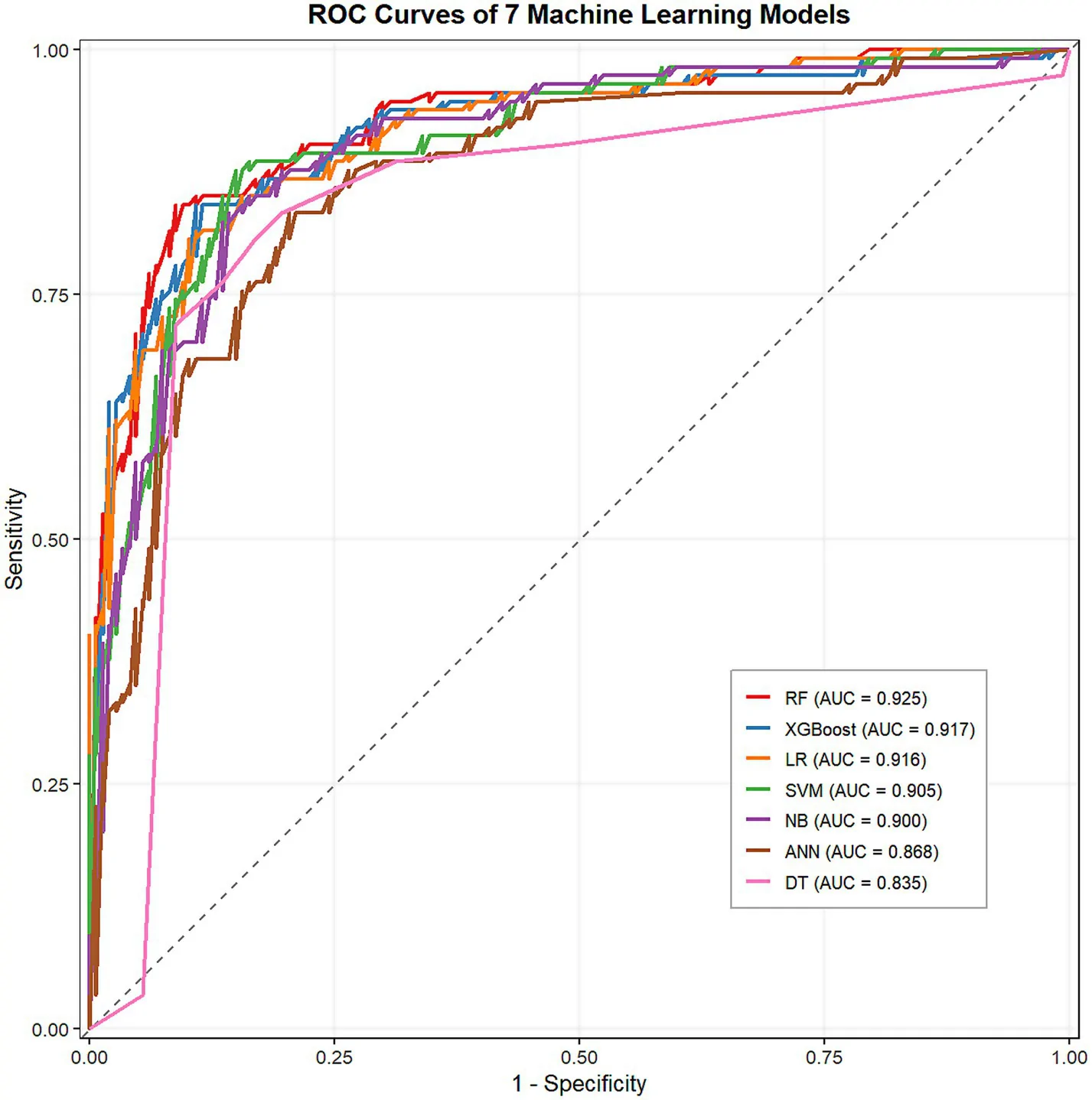
ROC curves for seven machine learning models predicting the risk of in-hospital mortality. AUC values are shown in the legend.

Ultimately, the average AUC from 10-fold cross-validation on the training set was selected as the primary selection criterion. The random forest model with the best cross-validation performance was chosen as the base model for subsequent feature pruning and interpretability analysis. Additional considerations included the fact that the random forest model demonstrated superior calibration and supported feature contribution analysis at both the global and individual levels using the SHAP method, which aligns more closely with the core objective of this study to construct an interpretable clinical prediction model.

### SHAP-based feature pruning and construction of the final 11-feature model

To improve the model’s conciseness and clinical utility, the SHAP algorithm was used to rank the feature importance in the 17-feature random forest model (see Supplementary Figure 1). Based on the average absolute SHAP values of the features, a sequential backward pruning strategy was employed to remove features over five iterative rounds, with the three least important features removed in each round. At each pruning step, model performance was re-evaluated using 10-fold cross-validation on the training set, and the cross-validation AUC (CV AUC) was recorded. The DeLong test was used to assess the statistical significance of changes in CV AUC (significance threshold: *p* < 0.05; relative AUC decline threshold: 2%). This iterative process continued until statistical significance was achieved or the AUC decline exceeded the preset threshold. The test set was set aside separately and was not accessed at any stage of the feature pruning process to ensure the fairness and unbiased nature of the final evaluation.

Figure 4 shows the ROC curves and corresponding AUC values for each feature subset model in 10-fold cross-validation on the training set, providing a visual representation of performance trends during the feature pruning process. It should be emphasized that all feature pruning and model selection decisions were based on the results of cross-validation on the training set; the independent test set was not involved in any stage of this process.

**Figure 4:**
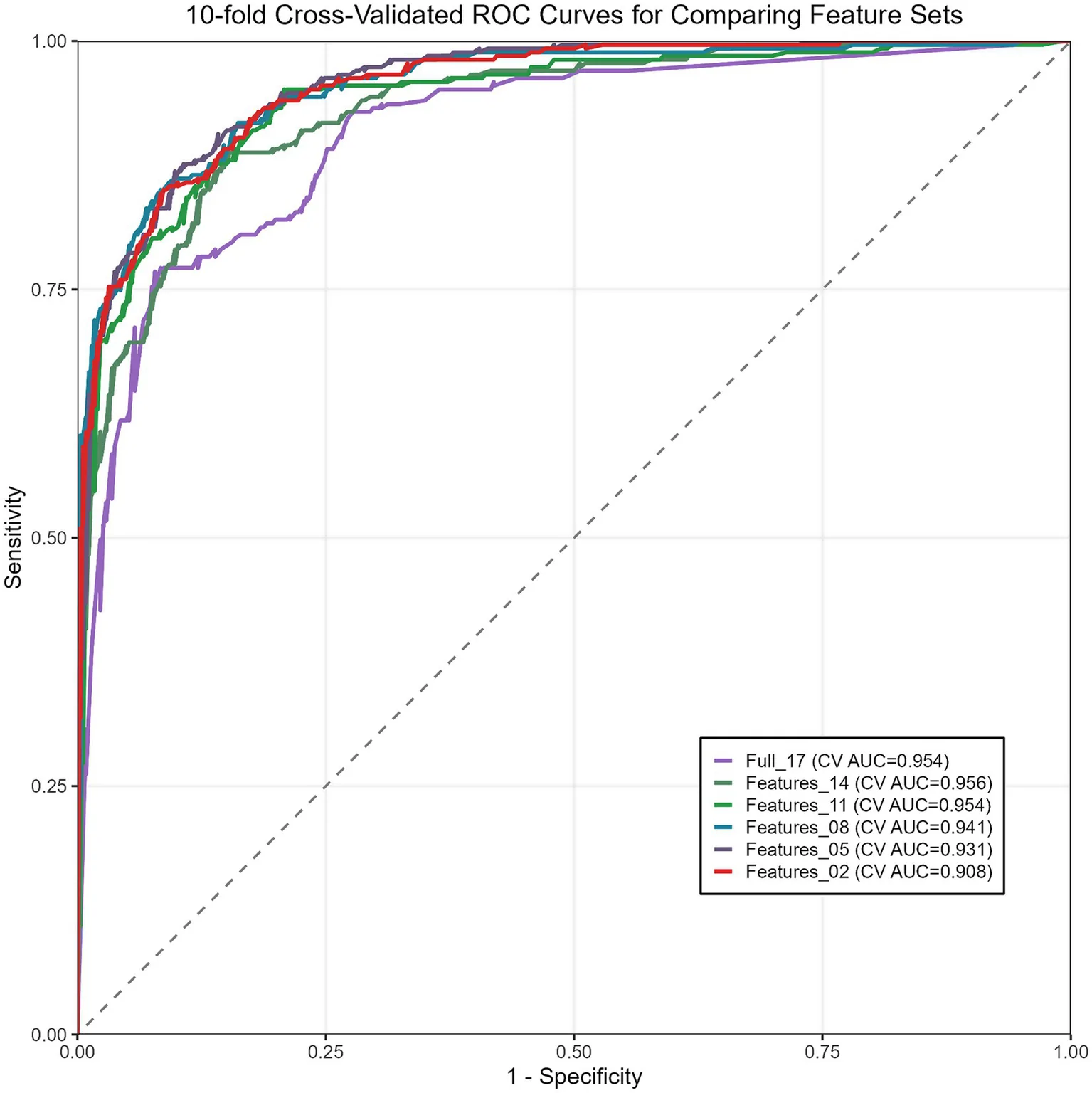
ROC curves–all feature reduction models. The AUC values for each feature subset shown in the figure are derived from independent test sets and are provided solely for illustrative purposes; they are not used in model selection or feature pruning decisions. All pruning decisions are based on the results of 10-fold cross-validation on the training set.

Supplementary Figure 2 illustrates the trend in the training set cross-validation AUC as the number of features changes. When the number of features was reduced from 17 to 11, the cross-validation AUC remained stable (17 features: 0.954; 14 features: 0.956; 11 features: 0.954), with no statistically significant decline (all *p* > 0.05). However, when the number of features was further reduced to 8, the CV AUC fell to 0.941, representing a statistically significant difference compared with the 11-feature model (DeLong test, *p* = 0.0013), thereby satisfying the stopping rule. Consequently, we ceased further feature pruning and ultimately selected the random forest model with 11 features as the final model.

This final model included the following predictor variables: GCS score, SOFA score, lactate, APACHE II score, FOUR score, pupil size, prothrombin time, midline shift, procalcitonin, diastolic blood pressure and oxygen saturation.

### Evaluation of the final model on an independent test set

The performance of the final 11-feature model was evaluated in a single assessment on an independent test set. The independent test set comprised a total of 261 patients, of whom 114 died in hospital (43.7%) and 147 survived (56.3%). The 2 × 2 confusion matrix for the final model is as follows: 97 true positives (TP), 16 false positives (FP), 131 true negatives (TN) and 17 false negatives (FN). Based on the above results, the following metrics were calculated: sensitivity 85.1 per cent, specificity 89.1 per cent, accuracy 87.4 per cent, precision 85.8 per cent, negative predictive value 88.5 per cent, and an F1 score of 0.854. Detailed values from the confusion matrix are listed in Supplementary Table 4.

### Evaluation of the calibration and clinical utility of the random forest model

We used calibration curves, quantitative calibration metrics, and the Hosmer-Lemeshow test to evaluate the calibration performance of this 11-feature reduced model on an independent test set. As shown in Figure 3A, the model’s calibration curve aligns well with the ideal diagonal line, and the observed mortality rates across each risk stratum generally correspond closely with the model’s predicted probabilities. Quantitative calibration metrics show that the model has a calibration slope of 1.047, an intercept of −0.093, a Dxy value of 0.847, and Brier Score: 0.103; these metrics reflect the overall level of prediction error and indicate that the goodness of fit is within the ideal range. The results of the Hosmer-Lemeshow test showed no statistically significant difference between the model-predicted probability and the actual in-hospital mortality rate among TBI patients (*p* = 0.499, *p* > 0.05). Taken together, these metrics indicate that the model has good calibration performance.

The results of the Decision Curve Analysis (DCA) are shown in Figure 5B. In this study, the range of 0 to 0.5 was selected as the core risk threshold evaluation interval; this choice was primarily based on the actual clinical decision-making context for severe TBI: This range corresponds to the conventional clinical decision-making range for in-hospital mortality risk in patients with severe TBI. In clinical practice, the decision thresholds for initiating interventions—such as escalating monitoring, implementing intracranial pressure (ICP) monitoring, dehydration to reduce ICP, or invasive surgery—largely fall within this risk range, making it the core segment for clinically balancing the benefits and risks of interventions. However, when the predicted risk threshold exceeds 0.5, patients are already classified as extremely high-risk, and clinical management pathways are relatively well-defined and standardized; thus, the incremental value of model-assisted risk stratification is limited. Consequently, the high-risk interval was not prioritized for evaluation. Within this interval, the net benefit of the 11-feature random forest model consistently exceeded that of the two control strategies “full intervention” and “no intervention.”

**Figure 5:**
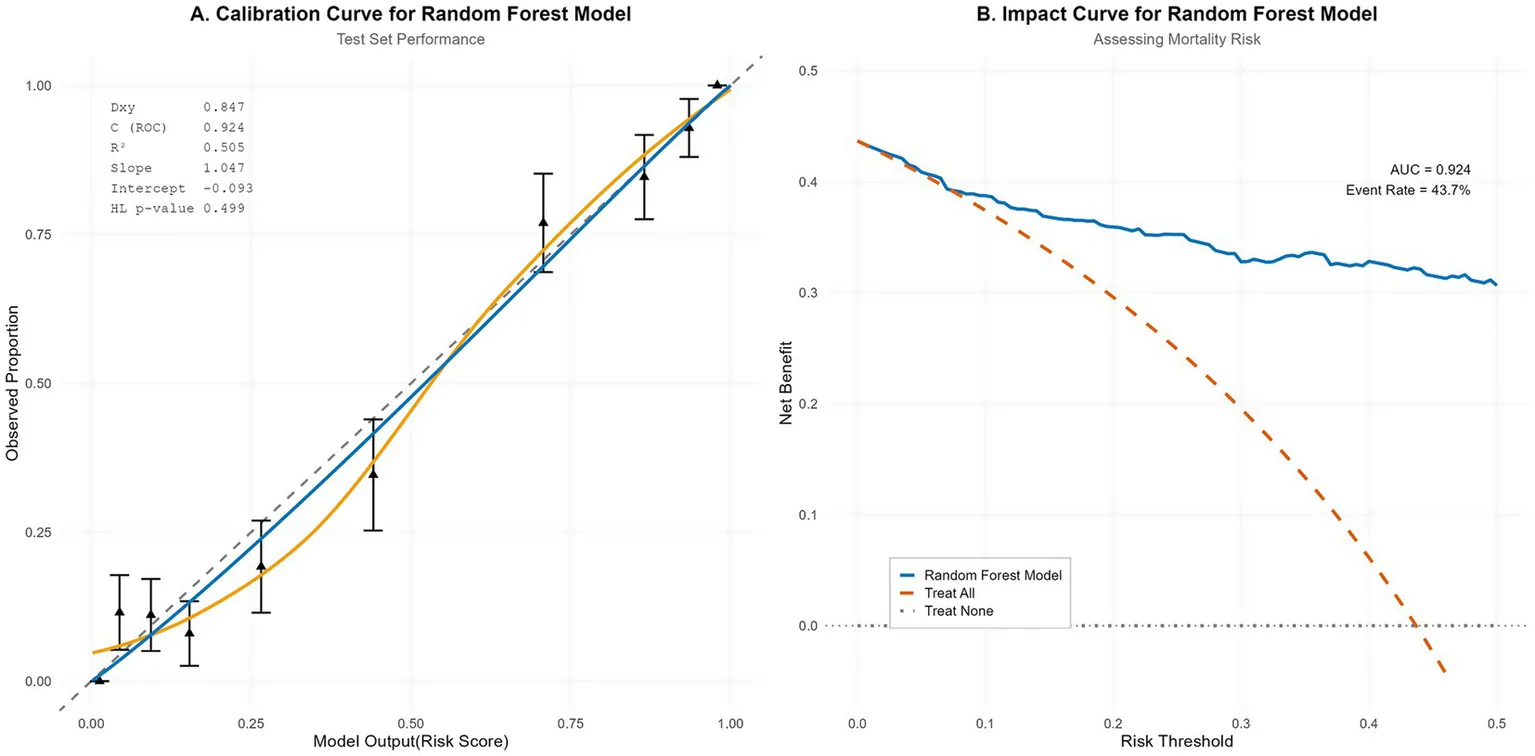
Calibration and decision curve analysis of the random forest prediction model for mortality. **(A)** Calibration curve in the test set, plotting the observed proportion against model-predicted risk score. Summary statistics including Dxy, C (ROC), R², slope, intercept and Hosmer-Lemeshow P-value are presented. **(B)** Decision impact curve illustrating net benefit across different risk thresholds. Three strategies are compared: the random forest model (blue solid line), treat all (orange dashed line), and treat none (gray dotted line). The model AUC is 0.924 and the overall event rate is 43.7%.

The above results indicate that the prognostic features identified in this study and the predictive model constructed demonstrate observable clinical net benefit in a retrospective cohort. However, since the decision curve analysis is based on retrospective data, it can only serve as a reference for potential clinical utility and does not represent the model’s actual performance in real-world clinical settings; its prospective application value still requires further validation through independent prospective studies (see Figure 5).

### SHAP-based model interpretability analysis

To enhance model transparency and clinical interpretability, this study conducted a SHAP interpretability analysis on the optimally pruned random forest model comprising 11 features.

The SHAP swarm plot (Figure 6) clearly illustrates the ranking of feature importance: the key features influencing the TBI-related in-hospital mortality risk in patients with severe traumatic brain injury (ranked from highest to lowest contribution) are, in order, the Glasgow Coma Scale (GCS) score, the Sequential Organ Failure Assessment (SOFA) score, lactate (lac), Acute Physiology and Chronic Health Evaluation II (APACHE II) score, Full Outline of Unresponsiveness (FOUR) Score, pupil size, prothrombin time (PT), Centerline Shift, procalcitonin (PCT), Oxygen Saturation (SpO₂), and diastolic blood pressure (DBP). Among these, the GCS score is the core predictive feature with the highest contribution to the model; The numerical values of each feature showed a clear correlation with the SHAP values, intuitively revealing the direction and strength of each variable’s contribution to the model’s decision-making process.

**Figure 6:**
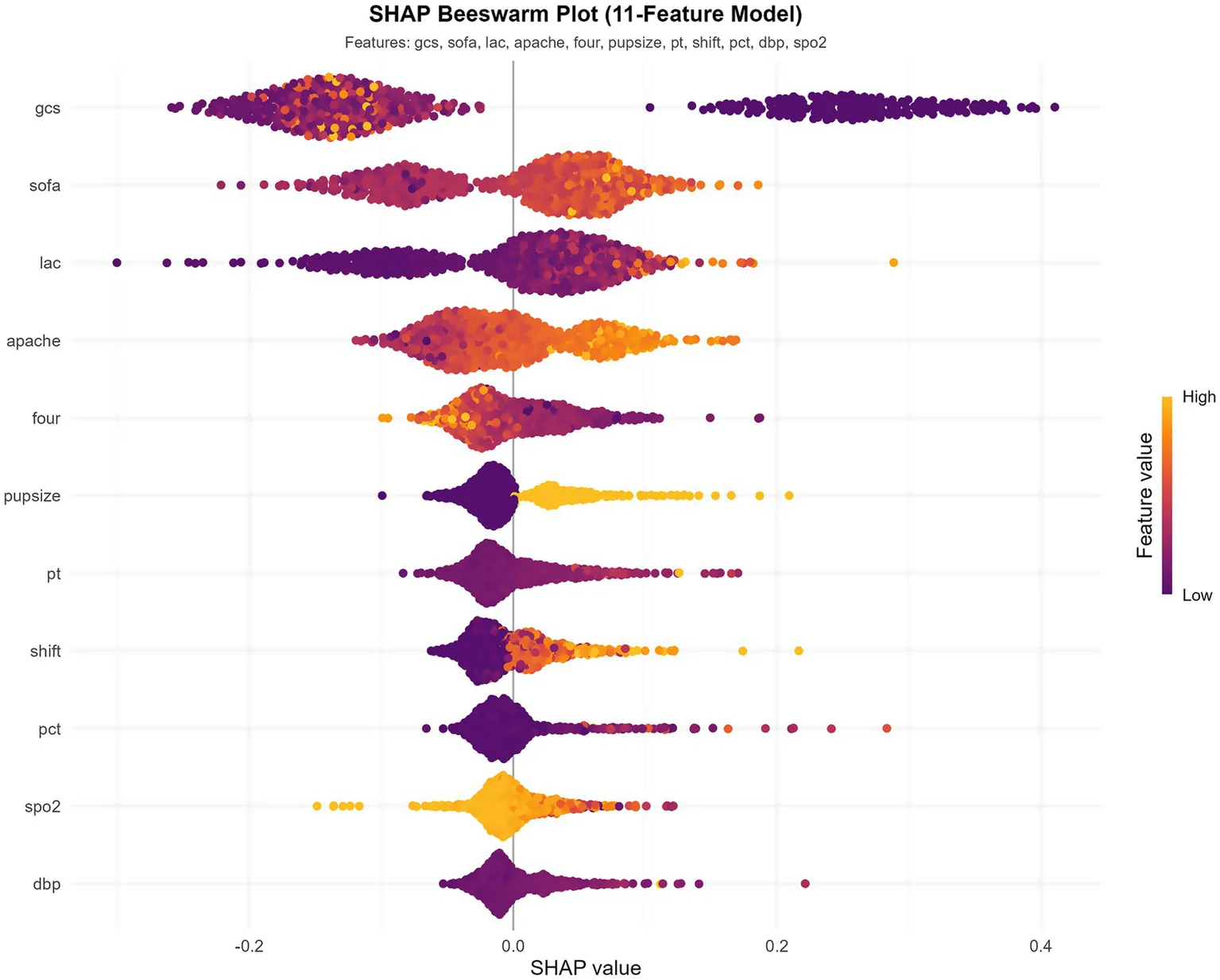
SHAP Beeswarm Plot (11-feature model). This plot displays the importance of the top 11 most significant predictors and the direction of their influence. Colors indicate feature value magnitude.

The SHAP dependency plot (Figure 7) further elucidates the nonlinear associations between each feature and the risk of death: the lower the GCS score, the higher the patient’s risk of death, and the model is more sensitive to changes in values within the GCS threshold range; higher lactate and procalcitonin levels are associated with a significant increase in the probability of death; Higher SOFA and APACHE II scores indicate more severe organ failure, and the risk of death increases accordingly; Dilated pupils, a low FOUR score, prolonged prothrombin time, and midline shift are all factors associated with an increased risk of death; decreased pulse oxygen saturation and abnormal diastolic blood pressure are also associated with an increased risk of death. Potential synergistic effects exist among some of these characteristics, and the aforementioned association patterns are consistent with clinical understanding and pathophysiological principles.

**Figure 7:**
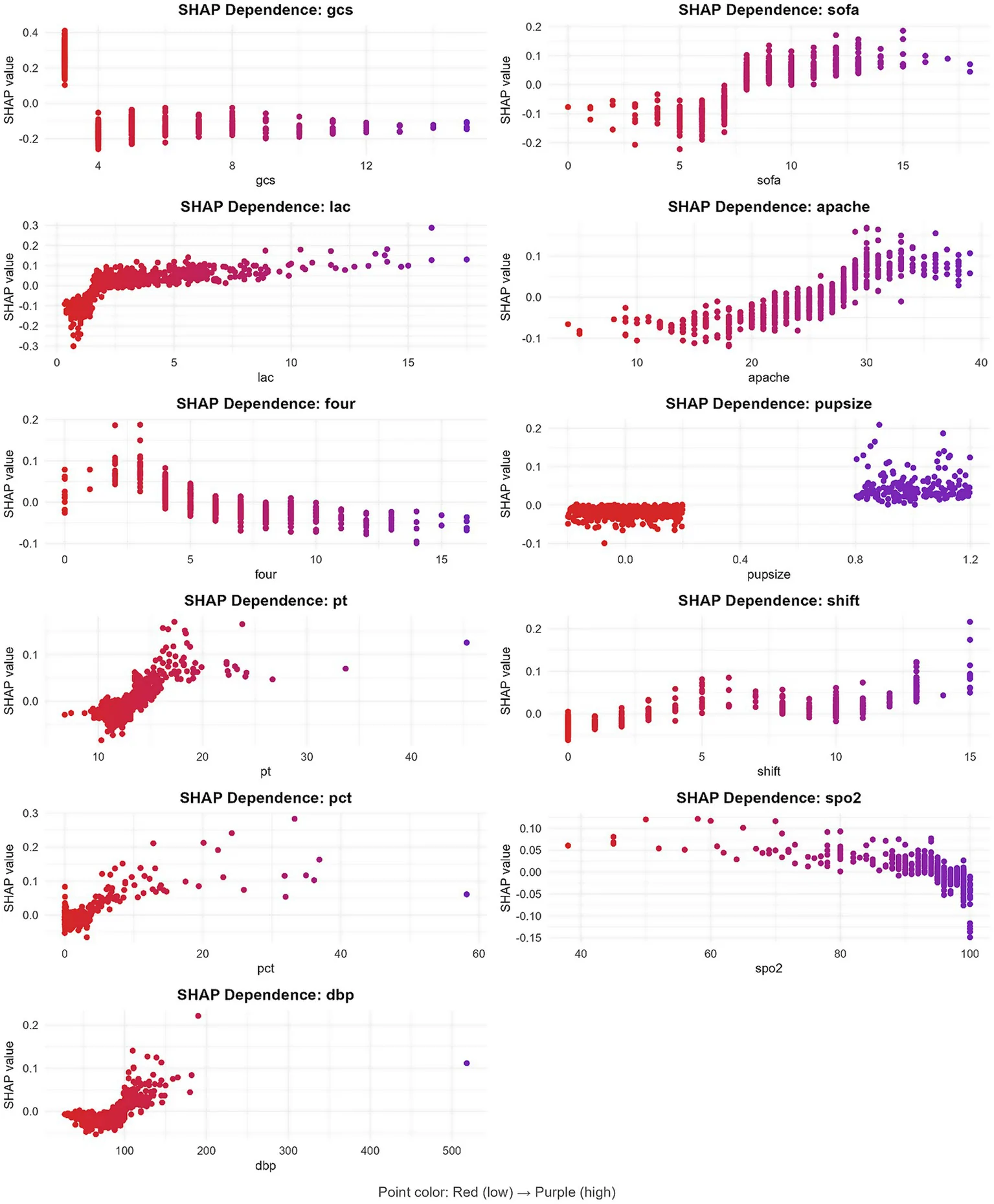
SHAP dependence plots for top 11 predictive features. This plot illustrates the impact of changes in feature values on the model’s predictive results. Point colors are graded by feature value, with red indicating higher values and blue indicating lower values.

In summary, the SHAP explainability analysis validated the clinical rationality of the model’s decision-making logic, providing a reliable and easily interpretable basis for the model’s clinical translation and application.

## Discussion

Traumatic brain injury is characterized by acute onset and rapid progression; given the complex nonlinear relationships among clinical indicators and individual heterogeneity, traditional single-score systems struggle to achieve precise stratification of mortality risk (37). These indicators spanned multiple dimensions, including neurological function, organ perfusion, coagulation function, and vital signs (38, 39). The results were consistent with those of previous studies (40, 41), laying the foundation for the development of a stable and reliable predictive model. Based on the selected features described above, this study systematically developed and compared seven machine learning algorithms. The evaluation results for the test set show that the Random Forest (RF) model had the highest point estimate of the AUC, at 0.923. Although Random Forest adds complexity compared with Logistic Regression, its better calibration and SHAP-based interpretability support its use in this study. In resource-limited settings, Logistic Regression may be a reasonable alternative given the comparable discriminative performance. To enhance the model’s clinical utility and reduce dimensional redundancy, this study utilized the SHAP algorithm to rank feature importance and perform stepwise feature pruning. Ultimately, a simplified model consisting of eleven core indicators was developed, achieving the optimal balance between model simplicity and clinical applicability while maintaining excellent predictive performance.

Based on a comprehensive evaluation of the calibration curve, quantitative calibration metrics (calibration slope, intercept, and Brier score), and the Hosmer-Lemeshow test, the 11-feature random forest model developed in this study demonstrated good calibration performance. The results of the Hosmer-Lemeshow test showed that there was no statistically significant difference between the model-predicted probability and the actual in-hospital mortality rate associated with TBI (*p* = 0.499, *p* > 0.05), suggesting the absence of significant systematic calibration bias and indicating that the model can provide a reliable probabilistic reference for clinical risk assessment. DCA shows that within the clinically common risk threshold range of 0 to 0.5, the model’s net benefit consistently exceeds that of the two control strategies: “full intervention” and “no intervention.” This threshold range corresponds to the common risk range for clinical decisions—such as initiating intensive monitoring and invasive interventions—in patients with severe TBI, and thus holds clear clinical significance; however, in the extremely high-risk range above 0.5, where clinical management pathways are well-defined, the model’s value for risk stratification is relatively limited. It should be noted that this analysis is based solely on retrospective data to evaluate the model’s potential clinical utility and does not represent its effectiveness in prospective applications; the model’s actual clinical benefits still require validation through subsequent prospective studies. This model provides a quantitative reference for clinical nursing risk stratification and is expected to assist nursing managers in optimizing resource allocation, promoting the precise and personalized distribution of medical resources, and aligning with the development goals of precision nursing and high-quality nursing services. In situations where nursing staff are in short supply, this model can also serve as a reference for the scientific allocation of nursing personnel, helping to improve the efficiency of resource allocation and supporting the core objective of advancing clinical nursing toward greater refinement.

Lack of interpretability is a major factor limiting the clinical translation of machine learning models. This study introduced SHAP explainability analysis, which to some extent overcomes the limitations of the “black box” nature of traditional machine learning models and quantifies the extent and direction of each feature’s contribution to the model’s mortality risk predictions. The results of this analysis provide clear priority rankings for clinical nursing assessment indicators and key monitoring dimensions, helping nursing staff identify core indicators closely related to prognosis and offering a reference basis for clinical condition assessment and risk stratification.

The Glasgow Coma Scale (GCS) is the predictor with the highest contribution in this model. SHAP analysis showed that lower GCS scores corresponded to higher positive SHAP values, indicating a higher risk of death. This finding is highly consistent with clinical principles of neurofunctional assessment centered on the level of consciousness (42), reflecting a direct correlation between the degree of impaired consciousness and the severity of brain injury. A rapid decline in the GCS score is often associated with acute worsening of intracranial conditions, suggesting that assessment of the level of consciousness is a top priority in the clinical monitoring of patients with TBI.

Lactate (Lac) is an important prognostic indicator; its SHAP value shows a marked upward trend as concentration increases, suggesting that lactate accumulation is closely associated with inadequate tissue perfusion. As a key marker of systemic hypoxia and metabolic disturbances, persistently elevated lactate levels indicate an imbalance in tissue oxygen supply and are associated with clinical deterioration and an increased risk of poor prognosis (43); therefore, lactate can serve as a key monitoring indicator of circulatory perfusion status in clinical assessments.

The SOFA score reflects the severity of multiorgan dysfunction; as the score increases, the corresponding SHAP value continues to rise, indicating that the more severe the organ failure, the higher the patient’s risk of death (44). Traumatic brain injury is often accompanied by systemic inflammatory response, circulatory instability, and respiratory dysfunction; a neurological assessment alone is insufficient to fully reflect the severity of the condition. The SOFA score encompasses functional assessments of multiple organ systems—including respiratory, circulatory, hepatic, and coagulation—and can serve as a reference for evaluating systemic risks associated with secondary injuries, thereby assisting healthcare providers in dynamically assessing the patient’s overall severity of illness.

As a classic comprehensive intensive care scoring system, the APACHE II score also makes a significant positive contribution to SHAP analysis. A higher score indicates a more severe disruption of overall physiological function and a higher risk of death (45). This index integrates acute physiological status with chronic health background and can assist in identifying high-risk patients with poor baseline health and weak compensatory capacity (41). In clinical nursing practice, the APACHE II score can be used to rapidly assess the overall severity of a patient’s condition, providing a reference for determining monitoring levels and the rational allocation of nursing resources.

The FOUR score, as a specialized assessment tool for neurocritical care, demonstrates clear prognostic value in the model. SHAP analysis shows that a lower FOUR score indicates more severe neurological impairment and a higher risk of mortality. Compared with the traditional Glasgow Coma Scale (GCS), the FOUR score provides a more detailed assessment of respiration, brainstem reflexes, and motor responses, making it more suitable for patients who cannot fully cooperate with GCS assessment due to sedation or endotracheal intubation (46). This suggests that the combined use of the FOUR score in the clinical assessment of complex neurocritical care cases can provide a more comprehensive risk assessment dimension.

Abnormal pupil size is associated with elevated intracranial pressure and an increased risk of brain herniation, which manifests in the model as a significant positive SHAP value. Pupillary dilation, bilateral asymmetry, or absence of the light reflex all indicate brainstem compression and constitute life-threatening clinical emergencies (47, 48). SHAP-dependent plots further demonstrate a clear gradient association between the severity of pupillary abnormalities and the risk of death, highlighting the important monitoring value of bedside pupillary assessment in evaluating the condition of TBI patients and providing a reference for clinicians to rapidly identify risks of critical illness.

Prolonged prothrombin time (PT) is significantly positively correlated with the risk of death, suggesting that coagulation dysfunction is associated with an increased risk of poor prognosis in patients with traumatic brain injury (TBI). Severe traumatic brain injury is often accompanied by coagulation activation, hyperfibrinolysis, and even disseminated intravascular coagulation, which can further exacerbate cerebral ischemia, hypoxia, and secondary injury (49). Model analysis showed that abnormal prothrombin time is positively correlated with the risk of death, suggesting that coagulation dysfunction is associated with an increased risk of in-hospital mortality in TBI patients and can serve as a key monitoring parameter for coagulation function in clinical condition assessment.

Midline shift showed a positive SHAP contribution in the model; the more pronounced the shift, the higher the risk of death. As a key radiological marker of the intracranial mass effect in severe TBI, the degree of midline shift is significantly associated with poor patient outcomes (50), and is one of the strongest independent prognostic factors on CT images. As a key radiological indicator reflecting the severity of intracranial mass effect, midline shift is directly related to the severity of the condition and can serve as a key monitoring parameter in the radiological assessment of patients with severe TBI.

In this model, procalcitonin (PCT) also exhibits a positive SHAP contribution; elevated levels of which indicate an increased risk of severe inflammatory response or secondary infection. Severe systemic inflammatory response syndrome following traumatic brain injury can lead to multi-organ dysfunction, while secondary infection can further exacerbate the clinical condition (51). As a biomarker for early infection and the severity of inflammation, PCT is associated with the risk of latent infection and the severity of inflammation, and can serve as a key indicator for monitoring infection-related conditions in clinical practice.

Blood oxygen saturation exhibits a negative SHAP contribution in the model; lower values indicate a higher risk of death. Hypoxemia following traumatic brain injury can exacerbate cerebral ischemia and hypoxia and worsen secondary brain injury, making it a significant risk factor for poor patient prognosis (52). As a basic vital sign reflecting the body’s oxygenation status, low oxygen saturation is associated with an increased risk of death and can serve as a core monitoring parameter in the routine vital sign monitoring of patients with TBI.

Diastolic blood pressure (DBP) showed a clear SHAP contribution in the model; abnormal fluctuations in diastolic blood pressure at admission were associated with an increased risk of mortality in TBI patients. Diastolic blood pressure is the core circulatory foundation for maintaining cerebral perfusion pressure; abnormalities in its levels can exacerbate secondary brain injury by affecting cerebral perfusion homeostasis and serve as an independent predictor of in-hospital mortality in TBI patients (28). As a key circulatory indicator for maintaining cerebral homeostasis, abnormal fluctuations in diastolic blood pressure are associated with the risk of clinical deterioration and can serve as a key monitoring dimension for assessing circulatory function in patients with severe TBI.

Hyperglycemia at admission is an independent risk factor for secondary brain injury and early mortality in patients with severe TBI; a study by Prisco et al. (53) also supports its role as a driver of adverse outcomes. Blood glucose was included in the initial pool of variables in this study; however, it did not make it into the final set of 11 core features following LASSO screening and stepwise pruning. This may be related to the fact that composite scores such as SOFA and APACHE II already partially capture information on systemic metabolic disturbances, thereby weakening the independent predictive contribution of blood glucose as a single variable.

In summary, the 11-feature explainable random forest model developed in this study demonstrates strong potential for clinical translation. This model supports predictive care by integrating multidimensional objective indicators, enabling nurses to identify high-risk patients early in emergency department and ICU settings, and facilitating a shift in nursing practice from reactive intervention to proactive early warning; The model’s clearly defined core observation indicators can be directly incorporated into nursing assessment checklists, thereby reducing bias in rule-of-thumb decision-making and promoting the standardization and systematization of nursing assessments. At the same time, the model has the potential for system integration; since it requires only eight routine clinical indicators, it can be easily embedded into electronic medical record systems or developed into intelligent decision-support tools to enable automated assessment and real-time alerts, offering significant practical value for improving the quality of care for patients with TBI.

## Study limitations and future directions

### Study limitations

When interpreting the results of this study, the following limitations should be carefully considered.

1. Inherent Limitations of the Study Design and Validation Strategy: This study was a two-center retrospective cohort study. Both centers were Level III trauma centers with highly consistent treatment protocols for severe TBI and a relatively homogeneous patient population in terms of baseline characteristics. The study employed an internal validation strategy using a combined 7:3 stratified random cohort; patients from the same hospital could be distributed across both the training and testing sets, making it impossible to verify the model’s cross-center generalization performance; Due to sample size limitations at a single center, “leave-one-center” external validation was not conducted to avoid compromising model stability due to insufficient sample size. Additionally, the retrospective design may introduce selection bias and recording bias. This study employed complete case analysis; however, because the original database did not systematically record missing values, it was not possible to report univariate missing rates or compare baseline characteristics between the included and excluded cohorts, which may further introduce selection bias.
2. The sample size provides limited support for machine learning methods: This study included a total of 874 patients. Although statistical power calculations indicated that the sample size was sufficient to support the development of a prognostic model, the sample size remains relatively limited for machine learning methods, which may restrict the model’s ability to capture subtle interactions among variables. The model’s external validity and generalizability still require validation through larger-scale, multicenter, prospective cohort studies.
3. Restrictions on the Model’s Applicability Due to the Prediction Time Point: This study uniformly set the baseline prediction time point at 24 h after ICU admission. Because complete 24-h baseline data could not be obtained, patients who died within 24 h of admission were excluded from the analysis, which may introduce survivor bias. Therefore, this model is applicable only to patients with severe TBI who were still alive 24 h after ICU admission; it does not cover those who died in the very early stages. Future research could explore the development of early prediction models based on data collected immediately upon ICU admission.
4. Limitations Regarding Population Coverage and Variable Dimensions: This study has not fully explored specific prognostic patterns in the elderly population with traumatic brain injury (TBI) (55), nor has it conducted a stratified analysis of elderly TBI patients. Furthermore, although previous studies (54) have demonstrated the significant value of the frailty index in predicting outcomes for older adults with traumatic brain injury (TBI), this model did not systematically incorporate this index into its predictive variables. Furthermore, the study did not account for potential confounding factors such as genetic polymorphisms, environmental differences, and variations in healthcare systems; the model’s discriminatory ability across different populations and clinical settings remains to be validated. In the future, integrating multidimensional healthcare data could enhance the model’s performance and adaptability to different populations.
5. Limitations Regarding Model Properties and Clinical Translation: This study developed a static predictive model that does not account for dynamic changes in patients’ clinical conditions. In the future, a dynamic predictive model based on time-series data could be developed to enable real-time monitoring and updating of the risk of in-hospital death. Furthermore, this study only completed model development and internal performance validation; it did not evaluate the model’s actual impact on clinical treatment decisions, cost-effectiveness, or real-world clinical value. The decision curve analysis, based solely on retrospective data, suggests potential clinical utility but does not represent the effectiveness of prospective application. Dedicated prospective studies are still needed to validate the model’s actual benefits before it is implemented in clinical practice.

### Future prospects

In the future, the interpretable predictive model developed in this study could be integrated with hospital electronic medical record systems to develop an intelligent clinical decision support system, enabling automatic assessment and real-time alerts regarding the risk of in-hospital mortality among TBI patients. At the same time, we will conduct prospective clinical studies to validate the model’s effectiveness and applicability, and calibrate and optimize the model based on the clinical characteristics of different centers to further enhance its generalizability. In addition, based on key model features, stratified intervention guidelines for in-hospital mortality risk in TBI patients can be developed to provide a reference for standardized clinical treatment, ultimately achieving the goals of reducing in-hospital mortality and improving clinical outcomes for TBI patients.

## Conclusion

The in-hospital mortality risk prediction model for patients with traumatic brain injury, developed in this study using a random forest algorithm, demonstrates good predictive performance. The 11-feature reduced model, derived through feature reduction and SHAP interpretability analysis, enhances clinical interpretability while maintaining predictive performance. It can effectively identify high-risk groups for in-hospital mortality and provides a simple and practical risk assessment tool for clinical nursing. The GCS, APACHE II score, SOFA score, FOUR score, lactate levels, pupil size, prothrombin time, midline shift, procalcitonin, diastolic blood pressure, and oxygen saturation are the core indicators identified by the 11-feature model in this study as being closely associated with the risk of in-hospital mortality in patients with traumatic brain injury. These are all routine assessment items that are clinically accessible and can be monitored dynamically; they align with the key focus areas of multidimensional nursing assessment. They provide data support for early risk stratification and individualized, targeted care, thereby helping to optimize proactive nursing and reduce decision-making bias. All the indicators required for this 11-feature simplified model are routinely available in ICU clinical practice; the model is easy to use and highly practical, making it suitable for routine prognostic risk assessment of patients with traumatic brain injury at the 24-h mark after admission to the intensive care unit (ICU). It can reduce the workload of nursing staff, support tiered care and the rational allocation of resources, and promote precise and standardized nursing practices. Future prospective studies are needed to further validate the model’s external validity, and to develop an intelligent clinical decision support system. Through precise risk assessment and targeted nursing care, this system aims to improve patient outcomes, reduce in-hospital mortality, and facilitate the implementation of high-quality nursing care.

## Data Availability

The raw data supporting the conclusions of this article will be made available by the authors, without undue reservation.
