# Supplementary material for: An interpretable machine learning model for predicting the risk of in-hospital death among ICU patients with traumatic brain injury
Source: Front Neurol. 2026 Oct 29;17:1987337. doi: 10.3389/fneur.2026.1987337 (PMC13754551; doi:10.3389/fneur.2026.1987337)
Supplement: Supplementary file 1 [file Supplementary_file_1.docx]

**Hyperparameter Settings and Grid Search Ranges for Machine Learning Models**

**Random Forest Hyperparameter Grid Search Range and Optimal Values**

mtry (number of variables selected for each split)

Search range: {2, 3, 4, 5, 6}

min.node.size (minimum sample size for leaf nodes)

Search range: {5}

splitrule (split rule)

Search range: {"gini"}

**Optimal Values**

mtry = 6

min.node.size = 5

splitrule = "gini"

**Validation Performance**

The 10-fold cross-validation accuracy on the training set was 88.6% (95% CI: 86.3–91.0).

**SVM Hyperparameter Search Range and Optimization Results**

**Hyperparameter Search Range**

cost (regularization parameter)

Search range: {0.1, 1, 5, 10, 20}

gamma (kernel parameter, sigma in caret)

Search range: {0.001, 0.01, 0.1, 1}

kernel (kernel type)

Search range: {"rbfdot"} (Radial Basis Function, RBF)

**Optimal Values**

cost = 20

gamma = 0.01

kernel = "rbfdot"

**XGBoost Hyperparameter Search Range and Optimization Results**

**Hyperparameter Search Range:**

nrounds (number of boosting rounds)

Search range: 500 (fixed with early stopping)

max_depth (maximum tree depth)

Search range: {3, 5, 7}

eta (learning rate)

Search range: {0.05, 0.1, 0.2}

gamma (minimum loss reduction)

Search range: {0, 0.1}

colsample_bytree (subsample ratio of columns)

Search range: 0.8 (fixed)

min_child_weight (minimum sum of instance weights)

Search range: 1 (fixed)

subsample (subsample ratio of training instances)

Search range: 0.8 (fixed)

**Optimal Values**

nrounds = 500

max_depth = 5

eta = 0.05

gamma = 0.1

colsample_bytree = 0.8

min_child_weight = 1

subsample = 0.8

Validation protocol: 10-fold cross-validation

**Decision Tree Hyperparameter Search Range and Optimization Results**

**Hyperparameter Search Range**

cp (complexity parameter)

Search range: {0.001, 0.003, 0.005, ..., 0.099}

minsplit (minimum observations for a split)

Search range: 20 (fixed)

minbucket (minimum data in one leaf)

Search range: 7 (fixed)

maxdepth (maximum tree depth)

Search range: 10 (fixed)

split rule

Search range: {"information"} (information gain / entropy)

**Optimal Values**

cp = 0.003

minsplit = 20

minbucket = 7

maxdepth = 10

split rule = information

**Naive Bayes Hyperparameter Search Range and Optimization Results**

**Hyperparameter Search Range**

laplace (Laplace smoothing)

Search range: {0} (fixed)

usekernel (kernel density estimation)

Search range: {TRUE, FALSE}

adjust (bandwidth adjustment for kernel density)

Search range: {1} (fixed)

**Optimal Values**

laplace = 0

usekernel = TRUE

adjust = 1

**Artificial Neural Network(ANN) Hyperparameter Search Range and Optimization Results**

**Hyperparameter Search Range**

size (number of hidden layer nodes)

Search range: {5, 10, 15, 20, 25}

decay (weight decay)

Search range: {0.001, 0.01, 0.1}

maxit (maximum iterations)

Search range: 500 (fixed)

**Optimal Values**

size (hidden nodes) = 25

decay (weight decay) = 0.001

maxit = 500
