# Supplementary material for: An interpretable machine learning model for predicting the risk of in-hospital death among ICU patients with traumatic brain injury
Source: Front Neurol. 2026 Oct 29;17:1987337. doi: 10.3389/fneur.2026.1987337 (PMC13754551; doi:10.3389/fneur.2026.1987337)
Supplement: Supplementary file 2 [file Table_1.docx]

| Supplementary Table 1. Demographic and Clinical Characteristics of Patients | | | | | |
| --- | --- | --- | --- | --- | --- |
| Characteristics | Subgroups | All patients (n=874) | Improved  (n=493) | In-hospital death (n=381) | P value* |
| gender | Male | 586(67.0) | 337(68.4) | 249(65.4) | 0.349 |
|  | Female | 288(33.0) | 156(31.6) | 132(34.6) |  |
| Previous stroke | No | 757(86.6) | 430(87.2) | 327(85.8) | 0.548 |
|  | Yes | 117(13.4) | 63(12.8) | 54(14.2) |  |
| History of hypertension | No | 501(57.3) | 296(60.0) | 205(53.8) | 0.065 |
|  | Yes | 373(42.7) | 197(40.0) | 176(46.2) |  |
| History of antihypertensive medication | No | 523(59.8) | 307(62.3) | 216(56.7) | 0.095 |
|  | Yes | 351(40.2) | 186(37.7) | 165(43.3) |  |
| History of diabetes mellitus | No | 738(84.4) | 422(85.6) | 316(82.9) | 0.282 |
|  | Yes | 136(15.6) | 71(14.4) | 65(17.1) |  |
| History of antidiabetic medication | No | 746(85.4) | 425(86.2) | 321(84.3) | 0.418 |
|  | Yes | 128(14.6) | 68(13.8) | 60(15.7) |  |
| History of atrial fibrillation | No | 800(91.5) | 455(92.3) | 345(90.6) | 0.359 |
|  | Yes | 74(8.5) | 38(7.7) | 36(9.4) |  |
| History of anticoagulant use | No | 776(88.8) | 444(90.1) | 332(87.1) | 0.175 |
|  | Yes | 98(11.2) | 49(9.9) | 49(12.9) |  |
| Pupil size | Abnormal | 589(67.4) | 194(50.9) | 395(80.1) | <0.001*** |
|  | Normal | 285(32.6) | 187(49.1) | 98(19.9) |  |
| Light reflex | Present | 587(67.2) | 323(84.8) | 264(53.5) | <0.001*** |
|  | Absent | 287(32.8) | 58(15.2) | 229(46.5) |  |
| Respiratory support | No | 69(7.9) | 59(12.0) | 10(2.6) | <0.001*** |
|  | Yes | 805(92.1) | 434(88.0) | 371(97.4) |  |
| Vasoactive agents | No | 242(27.7) | 171(34.7) | 71(18.6) | <0.001*** |
|  | Yes | 632(72.3) | 322(65.3) | 310(81.4) |  |
| Imaging type | CT | 853(97.6) | 477(96.8) | 376(98.7) | 0.099 |
|  | CTA | 18(2.1) | 14(2.8) | 4(1.0) |  |
|  | MRI | 2(0.2) | 2(0.4) | 0(0.0) |  |
|  | Others | 1(0.1) | 0(0.0) | 1(0.3) |  |
| Hematoma volume >30 mL | No | 576(65.9) | 371(75.3) | 205(53.8) | <0.001*** |
|  | Yes | 298(34.1) | 122(24.7) | 176(46.2) |  |
| Infarction range | <33% | 781(89.4) | 443(89.9) | 338(88.7) | 0.586 |
|  | >33% | 93(10.6) | 50(10.1) | 43(11.3) |  |
| Midline shift >10 mm | No | 579(66.2) | 367(74.4) | 212(55.6) | <0.001*** |
|  | Yes | 295(33.8) | 126(25.6) | 169(44.4) |  |
| Cerebral herniation | No | 544(62.2) | 354(71.8) | 190(49.9) | <0.001*** |
|  | Yes | 330(37.8) | 139(28.2) | 191(50.1) |  |
| Intraventricular hemorrhage | No | 447(51.1) | 258(52.3) | 189(49.6) | 0.424 |
|  | Yes | 427(48.9) | 235(47.7) | 192(50.4) |  |
| Age(years) |  | 62.00(53.00,70.00) | 62.00 (52.00,70.00) | 64.00(54.00,71.00) | 0.043 |
| Shift（mm） |  | 2.00 (0.00,10.00) | 0.00 (0.00,9.00) | 8.00 (0.00,11.00) | <0.001*** |
| Number of strokes |  | 0.00 (0.00,0.00) | 0.00 (0.00,0.00) | 0.00 (0.00,0.00) | 0.676 |
| Duration of hypertension（years） |  | 0.00 (0.00,6.25) | 0.00 (0.00,5.00) | 0.00 (0.00,8.00) | 0.053 |
| Duration of diabetes mellitus（years） |  | 0.00 (0.00,0.00) | 0.00 (0.00,0.00) | 0.00 (0.00,0.00) | 0.208 |
| Sbp（mmHg） |  | 149.00(120.00,177.00) | 149.00(124.50,175.00) | 148.00(110.00,180.00) | 0.398 |
| Dbp（mmHg） |  | 86.00 (72.00,100.00) | 86.00 (74.00,100.00) | 87.00 (68.00,103.00) | 0.822 |
| Hr（bpm） |  | 92.00 (77.00,113.00) | 89.00 (76.00,108.00) | 100.00 (80.00,120.00) | <0.001*** |
| Rr（bpm） |  | 18.00 (15.00,22.00) | 18.00 (15.00,22.00) | 18.00 (15.00,22.00) | 0.611 |
| Temp（℃） |  | 36.50 (36.50,36.80) | 36.60 (36.50,36.80) | 36.50 (36.50,36.80) | 0.010 |
| spo2（%） |  | 97.00 (93.00,99.00) | 97.00 (95.00,99.00) | 95.00 (90.00,99.00) | <0.001*** |
| GCS |  | 4.00 (3.00,6.00) | 5.00 (4.00,7.00) | 3.00 (3.00,4.00) | <0.001*** |
| FOUR |  | 6.00 (4.00,8.00) | 7.00 (5.00,8.00) | 4.00 (4.00,6.00) | <0.001*** |
| NIHSS |  | 0.00 (0.00,5.25) | 0.00 (0.00,10.00) | 0.00 (0.00,0.00) | 0.613 |
| APACHE Ⅱ |  | 25.00 (22.00,29.00) | 23.00 (19.00,26.00) | 29.00 (25.00,32.00) | <0.001*** |
| SOFA |  | 8.00 (7.00,10.00) | 7.00 (6.00,9.00) | 10.00 (8.00,12.00) | <0.001*** |
| Wbc（×10⁹/L） |  | 12.63 (9.60,16.57) | 12.08 (9.28,15.16) | 13.63 (10.24,17.83) | <0.001*** |
| Hgb（g/L） |  | 121.00(101.00,138.00) | 122.00(104.00,136.00) | 121.00(100.00,139.00) | 0.969 |
| Plt（×10⁹/L） |  | 176.00(132.00,221.00) | 176.00(136.00,224.50) | 175.00(125.00,217.00) | 0.077 |
| Pt（s） |  | 12.55 (11.50,14.00) | 12.30 (11.30,13.30) | 13.30 (12.00,15.15) | <0.001*** |
| Aptt（s） |  | 28.10 (25.50,31.50) | 27.40 (25.30,29.90) | 28.90 (26.05,34.30) | <0.001*** |
| INR |  | 1.15 (1.05,1.27) | 1.12 (1.05,1.21) | 1.20 (1.06,1.36) | <0.001*** |
| Ddimer（mg/L） |  | 3.44 (1.08,13.80) | 2.97 (0.86,8.80) | 6.01 (1.35,31.89) | <0.001*** |
| Na（mmol/L） |  | 140.00(137.00,143.00) | 140.00(138.00,143.00) | 141.00(137.00,144.00) | 0.282 |
| K（mmol/L） |  | 3.66 (3.30,4.00) | 3.70 (3.35,4.02) | 3.60 (3.20,4.00) | 0.074 |
| Cl（mmol/L） |  | 107.00(103.00,111.00) | 107.00(103.00,110.00) | 108.00(104.00,112.00) | <0.001*** |
| Bun（mmol/L） |  | 6.10 (4.80,7.80) | 5.90 (4.60,7.35) | 6.40 (5.10,8.70) | <0.001*** |
| ALT（U/L） |  | 22.00 (14.00,37.00) | 22.00 (14.00,35.50) | 21.00 (14.00,40.00) | 0.424 |
| AST（U/L） |  | 32.50 (23.00,53.00) | 31.00 (22.00,49.00) | 36.00 (24.00,59.00) | <0.001*** |
| Alb（g/L） |  | 35.00 (30.08,39.00) | 35.10 (31.00,39.40) | 34.70 (29.00,38.40) | 0.010 |
| ph |  | 7.39 (7.32,7.45) | 7.40 (7.35,7.46) | 7.36 (7.27,7.44) | <0.001*** |
| pao2（mmHg） |  | 127.00(90.35,182.00) | 126.00(91.00,181.00) | 128.00(87.00,183.00) | 0.865 |
| paco2（mmHg） |  | 38.00 (33.00,45.00) | 37.80 (33.00,43.35) | 39.00 (33.00,46.50) | 0.124 |
| be（mmol/L） |  | -1.80 (-5.03,1.80) | -0.90 (-3.60,2.30) | -3.40 (-6.35,0.45) | <0.001*** |
| lac（mmol/L） |  | 2.50 (1.40,4.40) | 1.80 (1.10,3.30) | 3.40 (2.10,5.85) | <0.001*** |
| pct（ng/mL） |  | 0.40 (0.07,1.57) | 0.27 (0.06,1.08) | 0.62 (0.11,2.80) | <0.001*** |
| crp（mg/L） |  | 27.50 (5.48,88.25) | 29.90 (6.75,89.50) | 25.00 (4.50,84.50) | 0.138 |

Notes:SBP, systolic blood pressure; DBP, diastolic blood pressure; HR, heart rate; RR, respiratory rate; Temp, temperature; SpO2, peripheral capillary oxygen saturation; GCS, Glasgow Coma Scale; FOUR, FOUR score; NIHSS, National Institutes of Health Stroke Scale; APACHE Ⅱ, Acute Physiology and Chronic Health Evaluation Ⅱ; SOFA, Sequential Organ Failure Assessment; WBC, white blood cell; Hgb, hemoglobin; Plt, platelet; PT, prothrombin time; APTT, activated partial thromboplastin time; INR, international normalized ratio; D-dimer, D-dimer; Na, sodium; K, potassium; Cl, chlorine; BUN, blood urea nitrogen; ALT, alanine aminotransferase; AST, aspartate aminotransferase; Alb, albumin; pH, potential of hydrogen; PaO2, partial pressure of arterial oxygen; PaCO2, partial pressure of arterial carbon dioxide; BE, base excess; Lac, lactate; PCT, procalcitonin; CRP, C-reactive protein.
