# Supplementary material for: An interpretable machine learning model for predicting the risk of in-hospital death among ICU patients with traumatic brain injury
Source: Front Neurol. 2026 Oct 29;17:1987337. doi: 10.3389/fneur.2026.1987337 (PMC13754551; doi:10.3389/fneur.2026.1987337)
Supplement: Supplementary file 3 [file Table_2.docx]

AUC and 95% Confidence Intervals of Machine Learning Models Based on 10-Fold Cross-Validation in the Training Set

| Model | CV_AUC | CV_CI_Lower | CV_CI_Upper |
| --- | --- | --- | --- |
| Random Forest | 0.9587 | 0.9438 | 0.9737 |
| XGBoost | 0.9572 | 0.9432 | 0.9712 |
| Decision Tree | 0.9297 | 0.9055 | 0.9538 |
| Naive Bayes | 0.9158 | 0.8938 | 0.9378 |
| SVM (RBF) | 0.9121 | 0.8899 | 0.9342 |
| Logistic Regression | 0.9085 | 0.886 | 0.9311 |
| Neural Network | 0.886 | 0.8597 | 0.9123 |
