# Supplementary material for: An interpretable machine learning model for predicting the risk of in-hospital death among ICU patients with traumatic brain injury
Source: Front Neurol. 2026 Oct 29;17:1987337. doi: 10.3389/fneur.2026.1987337 (PMC13754551; doi:10.3389/fneur.2026.1987337)
Supplement: Supplementary file 4 [file Table_3.docx]

| **Comparison** | **AUC_RF** | **AUC_Compare** | **Z** | **P_Value** |
| --- | --- | --- | --- | --- |
| RF vs XGBoost | 0.924 | 0.919 | 0.127 | 0.898 |
| RF vs Logistic Regression | 0.924 | 0.914 | 0.468 | 0.639 |

Supplementary Table3：11 Paired DeLong test comparing the AUCs of the feature-based random forest model and the control model on the test set
