# Supplementary material for: An interpretable machine learning model for predicting the risk of in-hospital death among ICU patients with traumatic brain injury
Source: Front Neurol. 2026 Oct 29;17:1987337. doi: 10.3389/fneur.2026.1987337 (PMC13754551; doi:10.3389/fneur.2026.1987337)
Supplement: Supplementary file 5 [file Table_4.docx]

Supplementary Table 4: Details on the Classification Performance of the 11-Feature Random Forest Model on the Test Set

| Metric | Value |
| --- | --- |
| True Positive (TP) | 97 |
| False Positive (FP) | 16 |
| True Negative (TN) | 131 |
| False Negative (FN) | 17 |
| Sensitivity (Recall) | 85.1% |
| Specificity | 89.1% |
| Precision | 85.8% |
| Negative Predictive Value (NPV) | 88.5% |
| Accuracy | 87.4% |
| F1 Score | 0.854 |
