# Supplementary material for: An interpretable machine learning model for predicting the risk of in-hospital death among ICU patients with traumatic brain injury
Source: Front Neurol. 2026 Oct 29;17:1987337. doi: 10.3389/fneur.2026.1987337 (PMC13754551; doi:10.3389/fneur.2026.1987337)
Supplement: Supplementary file 6 [file Table_5.docx]

Supplementary Table 5. Baseline characteristics and per-center validation performance of the 11-feature random forest model

| **Category** | **Indicator** | **Center A (n=598)** | **Center B (n=276)** |
| --- | --- | --- | --- |
| **Sample distribution** | Total sample size, n | 598 | 276 |
|  | Training set, n (%) | 419 (70.1) | 194 (70.3) |
|  | Test set, n (%) | 179 (29.9) | 82 (29.7) |
|  | In-hospital mortality, n (%) | 281 (47.0) | 100 (36.2) |
| **Key baseline characteristics** | Age, years, mean ± SD | 61.72 ± 13.54 | 58.04 ± 15.02 |
|  | GCS score, mean ± SD | 4.80 ± 2.23 | 5.36 ± 2.89 |
|  | APACHE II score, mean ± SD | 25.65 ± 5.74 | 23.86 ± 6.41 |
|  | SOFA score, mean ± SD | 8.92 ± 4.89 | 8.16 ± 2.86 |
|  | FOUR score, mean ± SD | 5.96 ± 2.67 | 6.48 ± 2.96 |
|  | Systolic blood pressure, mmHg, mean ± SD | 148.58 ± 44.27 | 152.63 ± 42.74 |
|  | Diastolic blood pressure, mmHg, mean ± SD | 86.47 ± 30.49 | 88.64 ± 22.31 |
|  | Oxygen saturation, %, mean ± SD | 93.66 ± 9.09 | 94.51 ± 6.78 |
|  | Lactate, mmol/L, mean ± SD | 3.43 ± 2.88 | 3.22 ± 2.86 |
|  | Procalcitonin, ng/mL, mean ± SD | 2.03 ± 5.88 | 1.90 ± 4.39 |
|  | Abnormal pupil size, n (%) | 203 (33.9) | 82 (29.7) |
|  | Abnormal pupillary light reflex, n (%) | 181 (30.3) | 106 (38.4) |
|  | Midline shift > 10 mm, n (%) | 208 (34.8) | 87 (31.5) |
| **Model performance** | AUC (95% CI) | 0.926 (0.905–0.948) | 0.951 (0.922–0.979) |
|  | Sensitivity, % | 84.7 | 92.0 |
|  | Specificity, % | 88.3 | 86.9 |

Note. No statistically significant differences were found in demographic characteristics, disease severity, core laboratory and imaging parameters between the two centers, indicating good data homogeneity. Samples from both centers were split into training and test sets at an approximate 7:3 ratio with balanced distribution. All performance metrics were calculated based on the independent test set of each center.
